# Cyclin D1 Induces Chromosomal Instability

**DOI:** 10.18632/oncotarget.476

**Published:** 2012-04-15

**Authors:** Mathew C. Casimiro, Richard G. Pestell

**Affiliations:** Department of Cancer Biology, Thomas Jefferson University & Hospital, Kimmel Cancer Center, Philadelphia, USA; Department of Cancer Biology and Medical Oncology, Thomas Jefferson University & Hospital, Kimmel Cancer Center, Philadelphia, USA.

Cyclin D1 was originally identified as a candidate oncogene activated in a subset of parthyroid tumors through genetic rearrangement [1]. We now understand that Cyclin D1 is a member of a family of cyclins that regulate progression through the cell cycle in a stepwise fashion from commitment to DNA replication through to cell division and cytokinesis [2]. Cyclin D1 binds the cyclin dependent kinases cdk4 and 6 to phosphorylate the retinoblastoma protein (RB) and initiate transition from G1 to S-phase. In addition to interacting with its principal substrate, RB, cyclin D1-Cdk4 acts on other substrates that have well defined roles within, carcinogenesis (Smad3) and mitochondrial function (Nrf1) [3]. There are a number of cyclin D1 functions that are independent of an associated kinase. Cyclin D1 is a modulator of co-regulators such as BRCA1 and nuclear receptors. Hulit et al was the first to show the abundance of cyclin D1 determines TF recruitment in the context of local chromatin, and did so *in vivo* [4]. Fu et al was the first to show cyclin D1 is recruited in the context of local chromatin, which in turn recruited chromatin modifying proteins (SUV39, HP1α, p300, HDAC 1, and HDAC 3) and altered the acetylation and methylation of chromatin associated histones [5]. Cyclin D1 thus regulates transcription at the chromatin level by interacting with histone deacetylases and various transcription factors to regulate genes that contribute to differentiation and proliferation [4]. Cyclin D1 promoter occupancy assessed by ChIP-ChIP technology mapped cyclin D1 to approximately 900 genes [6]. We extended these studies to the whole genome to map at high resolution, using ChIP-Seq, the global genomic footprint for cyclin D1 [7]. We identified 3,222 regions (intervals) associated with cyclin D1, approximately 70% of these intervals were within 10kb of 2, 840 genes with a high density located within 500bp of the transcriptional start point. We next investigated the transcription factor motifs enriched at the interval region and found the top hits included ERα, Sp1 and Ctcf. Interestingly Ctcf is a zinc finger DNA binding protein that regulates transcription, governs enhancer function and is involved in sister chromatid cohesion.

We next interrogated the functional pathways associated with the genes bound by cyclin D1. One of the most enriched terms was cell division; most of the genes being involved in G2/M phase and cellular mitosis. Increased abundance of cyclin D1 during G2/M has previously been described [8]. We used ChIP to verify that cyclin D1 bound the regulatory regions of genes involved in mitosis and QT-PCR to demonstrate that the gene transcripts were induced in cyclin D1 rescued *Ccnd1^−/−^* fibroblasts. Misregulation of genes that govern the mitotic phase often lead to chromosomal instability (CIN). Whether a cause or a consequence of tumorigenesis, CIN itself is recognized as promoting transformation, associated with poor prognosis and metastasis. Understanding the transcriptional role of cyclin D1 in promoting CIN is of considerable clinical importance since it is commonly over expressed in breast, pancreatic, lung cancer and lymphoma.

In *Ccnd1^−/−^* fibroblasts rescued with cyclin D1, the induction of polyploidy occurred in 3 cell division assessed by FACS analysis. In order to further classify the chromosomal abnormalities we employed spectral karyotyping (SKY), a whole genome painting assay that can recognize complex genomic rearrangements. Cyclin D1 induced aneuploidy in a relatively short amount of time and a large number of translocations, both reciprocal and nonreciprocal. Nonreciprocal translocations can be potently transforming since they can carry oncogenes at the breakpoint. A leading cause of aneuploidy is multipolar spindles caused by abnormal number or structure of centrosomes. In order to investigate the fidelity of the mitotic process we used high-resolution confocal microscopy to observe fibroblasts stained with markers of spindles (α-tubulin) and centrosomes (γ-tubulin). In cyclin D1 rescued *Ccnd1^−/−^* fibroblasts over 50% of the cells exhibited multiple centrosomes that give rise to increase multipolar spindles in prometaphase/metaphase. The abnormalities were also evident at the mitotic plate since measurements of the plate width were significantly increased in cyclin D1 rescued fibroblasts.

We developed mouse model systems to investigate the potential for cyclin D1 to induce CIN *in vivo*. In a mammary gland specific Tet-inducible model the acute expression profile regulated by cyclin D1 after 7 days was enriched in genes that rank highly with CIN. We also used a mammary gland targeted model (MMTV) to continuously express cyclin D1. The mice started to develop mammary gland tumors at 400 days and the tumor-free incidence was 40% in MMTV-cyclin D1. The gene expression profile of the tumors showed enrichment for the CIN signature. We next compared cyclin D1 expression and the highest ranking CIN genes to a breast cancer expression database and discovered that expression of genes promoting CIN are highly enriched in luminal subtype and that high cyclin D1 and CIN expression correlate specifically in the luminal B subtype. There is increasing interest in employing drugs in the clinic that exploit CIN in tumors. The high CIN expression index in luminal B breast cancer provides a basis for using Cdk and CIN inhibitors as a targeted therapeutic approach.
